# Design and experimental study of a rigid-flexible coupled back rehabilitation robot

**DOI:** 10.3389/fbioe.2026.1775122

**Published:** 2026-04-02

**Authors:** Delin Kong, Yage Wang, Fu Mo, Chunbao Wang, Zhiwei Huang, Ting Ouyang

**Affiliations:** School of Mechanical and Electrical Engineering, Guangdong University of Science and Technology, Dongguan, China

**Keywords:** back rehabilitation robot, compression spring support, human spine kinematics, lumbar motion analysis, rigid-flexible coupling, spine rehabilitation mechanism

## Abstract

**Background:**

Existing spinal rehabilitation mechanisms present several limitations. These include a restricted range of motion, low flexibility, and suboptimal comfort.

**Methodology:**

To overcome these issues, a novel rigid-flexible coupled spine parallel rehabilitation mechanism is proposed. The mechanism is supported by a compression spring to enhance adaptability and comfort. Initially, a simplified skeletal-muscular model of spinal motion was developed based on human spine analysis. Subsequently, a mathematical model describing the system’s kinematics was established.

**Results:**

Analysis of the model indicated that, under a 120 N force applied by the anterior deltoid fascicle, the mechanism exhibited a maximum deformation of 6.40 mm, meeting the design expectations. In both simulations and experimental tests of forward flexion, the maximum lumbar dorsal forward flexion angle reached 68.6°. The maximum lateral flexion angle achieved was 60.4°, while the maximum rotational angle reached 58.5°. The average maximum movement speed across different volunteers was 14.94°/s, closely aligning with the design target of 14.90°/s. Experimental measurements of the device’s activity angles showed averages of 37.6° for forward flexion, 13.56° for backward flexion, 13.62° for lateral flexion (left/right), and 17.5° for rotation (left/right). All measured values were within, or closely approximated, the design range targets.

**Conclusion:**

The study determined both the physiological movement range of the human spine and the effective working space of the proposed mechanism. The results confirm the rationality and effectiveness of the mechanism’s design.

## 1 Introduction

With China’s aging population and shifts in work and lifestyle, the incidence of lumbar diseases is rapidly increasing, increasingly affecting younger individuals (Chen et al., 2019; Chen et al., 2022). Prolonged sitting, bending, and flexing in incorrect postures can induce sustained lumbar muscle contraction. This often leads to muscle fatigue and stiffness, increasing the mechanical load on the lumbar spine and elevating the risk of lumbar disorders. Effective lumbar rehabilitation training can alleviate neurological deficits, prevent excessive back muscle contraction, and improve patients’ motor functions (Yang and Mao, 2023). The spine is a complex structure composed of cervical, thoracic, lumbar, and sacral vertebrae, along with associated muscles and tendons (Aquino et al., 2019). In daily activities, the spine exhibits substantial flexibility, enabling motion such as flexion, extension, lateral bending, and rotation. Movements of the upper and lower extremities are coordinated through the spine, facilitating the execution of a wide range of functional activities (Zhang et al., 2020). Lumbar spinal stability is a critical determinant of overall spinal health and a primary target in the treatment of lumbar disorders. This stability relies on the coordinated interaction of the osteoarticular structures, lumbar musculature, and the spine’s physiological curvature (Liang et al., 2022; Liu et al., 2024). Therefore, investigating spinal joint rehabilitation mechanisms in back exoskeleton rehabilitation robots is essential for advancing lumbar therapy (Tian et al., 2023).

The motion axes of traditional rigid exoskeleton rehabilitation robots are usually fixed. As a result, the device can become misaligned with human joints, generating significant impact forces during sudden movements, which is detrimental to patient rehabilitation. To address this issue, researchers worldwide have explored lumbar rehabilitation robots with varied rehabilitation modes. For instance, Guo et al. (2020) designed a wearable 3-RPR parallel lumbar rehabilitation robot. Li et al. (2023) developed a novel wearable back-support exoskeleton. Tadano et al. (2019) designed an active thoracolumbosacral orthosis using two tandem Stewart-Gough platforms. Al-Dahiree et al. (2022) proposed a compact lumbar spine support exoskeleton robot, while Kim et al. (2021) designed an anti-gravity lumbar rehabilitation robot employing elastic actuators. These mechanisms are effective at compensating for joint misalignments. However, they have limitations, including reduced precision in exoskeleton control, inconvenient donning and doffing, added weight on the body, and decreased back muscle activation. Rigid-flexible coupling mechanisms offer advantages such as low motion inertia, adaptability, suppleness, and a large range of motion (Li et al., 2022; Zheng et al., 2024). Applying a rigid-flexible coupled parallel mechanism to lumbar spine rehabilitation can effectively eliminate misalignment between the rehabilitation device and the human lumbar joints. In recent years, rigid-flexible coupled rehabilitation mechanisms have seen wide application in robotics. For example, Niyetkaliyev et al. (2021) designed a five-degree-of-freedom (DoF) hybrid robot combining rigid rods and ropes. Tian et al. (2023) utilized bellows-type flexible actuators to develop a compact, home-use lumbar rehabilitation robot. You et al. (2024) designed a small home lumbar rehabilitation robot based on under-drive and varicell principles, incorporating a rigid-flexible coupled varicell hand claw. Zhang et al. (2024) designed a 6-DoF upper-limb rehabilitation robot to evaluate locomotor capabilities. Ma et al. (2022) proposed a 3-DoF rigid-flexible coupled parallel ankle rehabilitation mechanism, and Wang et al. (2019) designed a two-DoF rigid-flexible hybrid-drive ankle rehabilitation robot.

Numerous studies have examined the kinematics, optimal positioning, and workspace of rigid-flexible coupled mechanisms. Duan et al. (2016) proposed a rope-spring mechanism and analyzed how spring parameters influence its feasible workspace. Li et al. (2019) conducted a numerical study on the dynamics of a rigid-flexible coupled knee exoskeleton, based on sagittal gait planning. In a connected study, Ma et al. (2017) designed a rigid-flexible coupled parallel mechanism and performed a kinematic position analysis. Zhang et al. (2022) introduced a 3D spatial two-chain rigid-flexible coupled manipulator system with time-varying disturbances and input constraints, establishing its kinematic model. Building on a multi-rigid-flexible body dynamics inverse solution, Zhou et al. (2024) proposed a multi-DoF, bionic, and unpowered lumbar exoskeleton robot that incorporates key human biomechanical properties. Therefore, when simulating lumbar spine force deformation under simplified loading conditions, it is essential to include flexible components in the rehabilitation robot. These components should represent the soft tissues of the human body, such as muscles, ligaments, and intervertebral discs, and are crucial for precise control of the dynamic characteristics of the rigid-flexible coupled lumbar spine kinematic load-bearing mechanism proposed in this study (Panico et al., 2021; Xie et al., 2021; Wang et al., 2021).

However, several key challenges remain. First, many existing devices focus on actuation and control without fully integrating bio-inspired, passive flexible elements that accurately replicate the viscoelastic support of the vertebral-disc-ligament complex. Second, although rigid-flexible couplings are recognized as beneficial, designing a simple yet effective flexible spine analogue within a parallel structure, specifically for back rehabilitation, remains underexplored. Finally, there is a need for mechanisms that not only ensure kinematic compatibility but also provide inherently safe, spring-like support and motion damping in a compact form suitable for rehabilitation training. To address these requirements, this paper proposes a novel flexible parallel lumbar rehabilitation mechanism. The mechanism is driven by a moving rod and supported by a compression spring, inspired by the skeletal muscle anatomy of the spinal joints.

The compression spring mimics the supportive function of the lumbar spinal complex, while the moving rod replicates the action of the back muscles. This biomimetic design enables three-DoF in spinal rehabilitation movements. A simplified model of the spinal bone-muscle structure is established through an analysis of human spine anatomy. Based on this, a mathematical model of the system’s kinematics is constructed. Using the mechanism model, numerical simulations of back motion and posture under different loads are performed. Simulation and experimental testing provide the physiological motion range of the human back and the effective working space of the mechanism, verifying both the design rationality and the accuracy of the analysis method. Finally, the system was validated with a healthy volunteer. The proposed methodology can be extended to other parallel mechanisms supported by flexible components, providing a theoretical foundation for the design of rehabilitation robots.

## 2 Materials and methods

### 2.1 Design of the rigid-flexible coupled parallel rehabilitation mechanism

#### 2.1.1 Rigid-flexible spine mechanism analysis

**FIGURE 1:**
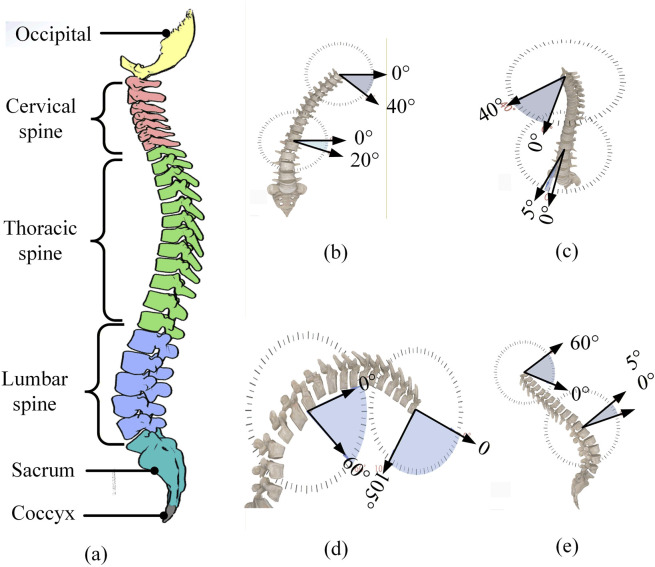
Physiological structure of the spine and its range of motion. **(a)** Schematic diagram of the spinal column. **(b)** Lateral flexion exercise. **(c)** Rotation exercise. **(d)** Forward flexion exercise. **(e)** Back extension exercise.

The adult human spine consists of 26 vertebrae, connected by ligaments, joints, and intervertebral discs. The lumbar region allows triaxial rotation around the coronal, sagittal, and transverse axes, enabling flexion/extension, anterior/posterior flexion, and rotation of the lumbar vertebrae on the left and right sides (Heo et al., 2020), as illustrated in Figure 1.

The lumbar spine plays a critical role in the human body, supporting the weight of the upper body, maintaining posture, and cushioning movements during strenuous activities. As the most weight-bearing segment of the spine, the lumbar region is also highly susceptible to strain (Zuo et al., 2014). Due to ligament constraints, vertebral motion involves both rotation and slippage, causing the axis of the vertebral joints to change continuously during movement. To address motion matching between the rehabilitation mechanism and the human spine, and to ensure patient safety, the motion patterns of the lumbar and thoracic vertebral joints are analyzed based on musculoskeletal bio-coupling characteristics. Back muscle groups are simplified, and their associated ligaments are further subdivided to model the vertebral bone-disc-ligament complex (Wei, 2017; Li et al., 2022). The motion of the lumbar and thoracic vertebral bone-disc-ligament complex is simplified for modeling the spine. This simplification is intended to safely and effectively restore the patient’s normal lumbar physiological curvature. As a result, a lumbar rehabilitation robot requires an upper surface with variable curvature to accommodate the lumbar spine at different stages of rehabilitation. Its height and angle must also be adjustable to ensure proper alignment with the human lumbar region (Wang, 2023). Notably, springs, as a type of assistive element, have been widely employed in exoskeleton devices to provide necessary adaptability (Zhang et al., 2016; Kazerooni et al., 2019; Han et al., 2020; van Sluijs et al., 2023).

#### 2.1.2 Design of a flexible parallel back section rehabilitation mechanism

**FIGURE 2:**
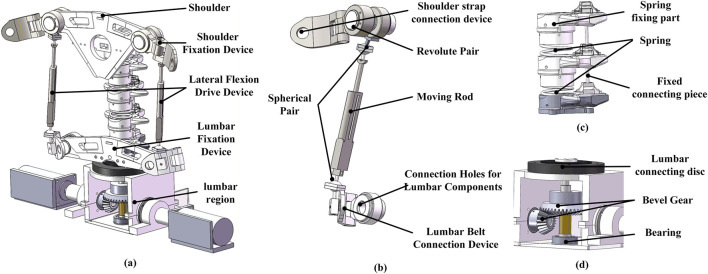
Parallel back rehabilitation mechanism for the rigid-flexible coupled spine. **(a)** Overall Structure of the Flexible Parallel Lumbar Rehabilitation Mechanism. **(b)** Flexible parallel drive rod. **(c)** Rigid-flexible spinal column model. **(d)** Rotary drive device.

This paper presents a novel flexible parallel back section rehabilitation mechanism, driven by a moving rod and supported by a compression spring. To enable the human lumbar spine to perform three degrees of freedom movements, the spring-supported spine mechanism is actuated by a parallel mobile rod system. The mechanism comprises three main components: a shoulder fixation device, a rigid-flexible parallel mechanism, and a lumbar fixation device. The model of the rigid-flexible spine rehabilitation mechanism is illustrated in Figure 2.

Due to variations in the distances between the axes of different lumbar spine injuries and the natural extension, flexion, and rotation axes of the spine, some patients, particularly those with severe hemiplegia, require their backs to be secured to the rehabilitation device using straps. This can further increase the distance between the two axes. To accommodate these differences, the shoulder fixation device is adjustable, allowing the distance between the two axes to be tailored to individual patients. This ensures that patients can perform rehabilitation exercises comfortably while minimizing the risk of secondary injury. An attitude sensor is installed in the front fixation device, allowing precise measurement of the moving platform’s orientation during the rehabilitation process. To conform to the curvature of the human back, the shoulder-lumbar fixation device features a front-mounted and rigid-spring-connected spine design, enhancing patient comfort during use. The shoulder fixation device and lumbar fixation device are linked via a rigid-flexible parallel mechanism. Within the flexible parallel mechanism, the moving platform simulates the scapula end, while the fixed platform represents the sacrum and hip bones. The rigid-spring connection emulates the lumbar vertebrae, intervertebral discs, and ligament complexes, providing support for the moving platform and decelerating the device during motion. This configuration replicates the functions of the lumbar vertebral joints and intervertebral disc joints. Finally, the two moving rods and motor at the end of the platform simulate muscular action, driving the flexible parallel rehabilitation mechanism.

### 2.2 Kinematic modeling

**FIGURE 3:**
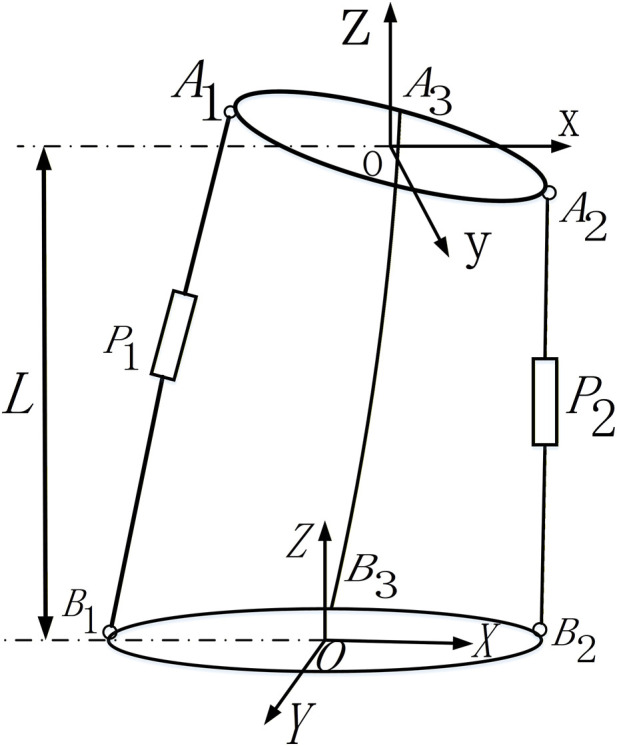
Schematic diagram of the rigid-flexible parallel mechanism. [P1, P2: Active adjusting moving rod mechanisms; L1: Moving rod of the P1 adjusting mechanism (connecting B1 and A1); L2: Moving rod of the P2 adjusting mechanism (connecting B2 and A2); L3: Rigid body-spring unit (connecting B3 and A3); O-XYZ: Global coordinate system of the parallel mechanism; o-xyz: Local coordinate system of the parallel mechanism; Fixed platform: Simulating the sacrum and hip bone; Moving platform: Simulating the scapula].

The analysis of the mechanism’s kinematic parameters forms the foundation for achieving precise control, taking into account the physiological structure of the human waist and its bending load-bearing biomechanical properties (Panico et al., 2021). The flexible parallel mechanism investigated in this study primarily consists of a composite assembly with four main components: the fixed platform, moving platform, moving rods, and a rigid body-spring unit. A schematic diagram of this rigid-flexible parallel mechanism is shown in Figure 3. The mechanism’s four core elements include the fixed platform, moving platform, active adjusting moving rods (P1 and P2), and the rigid body-spring unit (L3). The fixed platform represents the human sacrum and hip bones, while the moving platform simulates the scapula end. P1 and P2 serve as active drive components enabling lateral flexion motion, whereas L3 provides flexible support, emulating the lumbar vertebra-disc-ligament complex. Spherical hinge connection points (A1-A3, B1-B3) allow flexible articulation between each component. Global and local coordinate systems are used to characterize the mechanism’s spatial position and motion attitude, respectively.

With referring to Figure 3, let the positive direction of OY represent forward flexion motion, and the directions of OBi (i = 1, 2) represent left and right lateral flexion. Define O-XYZ as the global coordinate system of the parallel mechanism, with the origin O at the geometric center of the fixed platform. The X-axis is along the OB2 direction, the X-axis is perpendicular to the Y-axis, and the Z-axis is upward, perpendicular to the plane of the fixed platform. A local coordinate system, o-xyz, is established on the moving platform, with its origin O at the geometric center of the moving platform. In this local coordinate system, the y-axis is along the oA2 direction, the x-axis is perpendicular to the y-axis, and the z-axis is upward, perpendicular to the plane of the moving platform. One end of the active adjusting moving rods (P1 and P2) is connected to ball sub Ai (i = 1, 2) fixed to the moving platform, and the other end is connected to ball sub Bi (i = 1, 2) on the fixed platform. The fixed platform end of each rod is also connected to the output of the drive motor. The rigid body-spring unit is fixed at point A3 on the moving platform and at point B3 on the fixed platform. When the mechanism is unloaded, OAi and oBi are parallel, and Ai and Bi are equidistant along circles with radii |OBi| = b and |oAi| = a, respectively. Four parameters describe the motion of the moving platform: α is the angle between the Z_z_-axis and the X-axis, indicating left-right lateral flexion of the moving platform; β is the angle between the Zz-axis and the Y-axis, representing anterior-posterior flexion of the moving platform; L is the vertical distance from the origin of the moving platform to the plane of the fixed platform; and γ is the angle between the x-axis of the moving platform and the X-axis of the fixed platform, representing spinal column rotation.

Under the global coordinate system O-XYZ, the chi-square coordinate transformation matrix of points B_i_ on the fixed platform can be expressed as: B1 = [-b, 0, 0, 1], B2 = [b, 0, 0, 1], B3 = [0, -b, 0, 1], and the chi-square coordinates of points A_i_ on the moving platform are: A1 = [-a, 0, 0, 1], A2 = [a, 0, 1], A3 = [0, -a, 0, 1]. The rotation matrix, derived from Z-Y-X Euler angles, is used to obtain the chi-square transformation matrix from the local coordinate system of the moving platform to the global coordinate system (Equation 1):

ToO=r11r12r130r21r22r230r31r32r33L0001 (1)

Based on the established kinematic parameters of the rigid-flexible parallel mechanism, the relationships between these parameters are further deduced. According to the mechanism’s geometric structure and kinematic characteristics, under the global coordinate system O-XYZ, each point Ai on the moving platform is connected to the corresponding point Bi on the fixed platform by a moving rod. The length of each moving rod, L_i_, determines the motion state of the moving platform.

From the geometric relationships, the connection between the moving rod length Li and the moving platform motion parameters α, β, L , and γ can be expressed as follows (Equation 2):

$$L_{i}=\sqrt{{\left(x_{Ai}-x_{Bi}\right)}^{2}+{\left(y_{Ai}-y_{Bi}\right)}^{2}+{\left(z_{Ai}-z_{Bi}\right)}^{2}}$$ (2)

Here, ( $x_{Ai}$ , $y_{Ai}$ , $z_{Ai}$ ) and ( $x_{Bi}$ , $y_{Bi}$ , $z_{Bi}$ ) represent the coordinates of point Ai and point Bi under the global coordinate system, respectively.

By substituting the chi-square coordinates of points Ai and points Bi into the above equation and applying the Euler angle rotation matrix for coordinate transformation, the following relationships are obtained (Equations 3–5):

$$L_{1}=\sqrt{{\left(-a\cos\gamma \cos\alpha +b\right)}^{2}+{\left(-a\cos\gamma \sin\alpha \right)}^{2}+{\left(a\sin\gamma -L\right)}^{2}}$$ (3)

$$L_{2}=\sqrt{{\left(a\cos\gamma \cos\alpha -b\right)}^{2}+{\left(a\cos\gamma \sin\alpha \right)}^{2}+{\left(a\sin\gamma -L\right)}^{2}}$$ (4)

$$L_{3}=\sqrt{{\left(-a\sin\gamma \right)}^{2}+{\left(-a\cos\gamma -b\right)}^{2}+{\left(a\sin\gamma -L\right)}^{2}}$$ (5)

**FIGURE 4:**
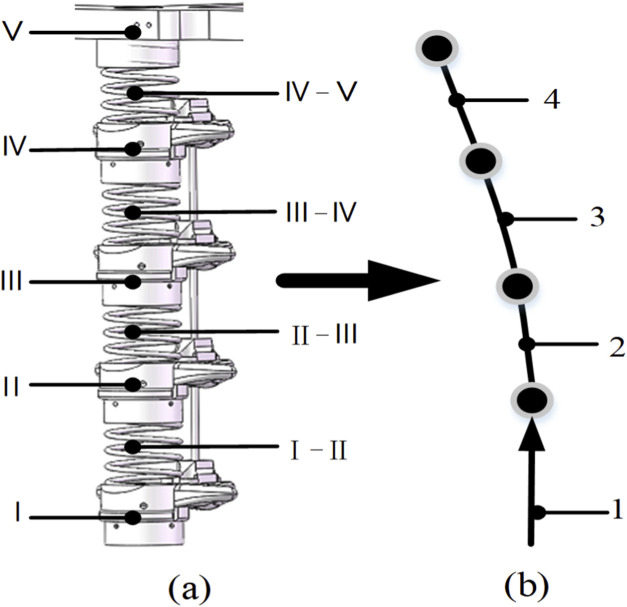
Sagittal plane equivalent sketch of the exoskeleton robot. **(a)** Rigid-body spring structure. **(b)** Equivalent mode of the lumbar spin.

The exoskeleton’s solid components are labeled I through V. The segments connecting these components (I-II, II-III, III-IV, and IV-V) represent equivalent spring elements, modeling the human spinal discs. The numbering convention follows the coordinate system; for example, the composite unit I - (I-II) - II represents an equivalent lumbar vertebra structure. This configuration is illustrated in Figure 4a. Considering the mobility of the human thoracic spine, all driving thrust and pull forces can be represented as an equivalent force and equivalent moment applied at the top center of the spring. The flexible members are labeled 1-4, as shown in Figure 4b, though their dynamics will not be discussed further.

Consequently, the position vector r_i_ (Han et al., 2016) at any point on the rigid-spring connection member can be expressed as Equation 6:

ri=Ri+TOoSijq¯oij+q¯fij (6)

In the formula, the upper horizontal line indicates that the vector is measured in the corresponding body coordinate system, while the absence of the upper horizontal line denotes measurement in the global coordinate system. $R^{i}$ represents the position vector of the origin of the component’s body coordinate system. ${\bar{q}}_{o}^{ij}$ and ${\bar{q}}_{f}^{ij}$ are the elasticity coordinates of the nodes of the component unit before and after deformation, respectively. TOo is the attitude transformation matrix of the local coordinate system o-xyz relative to the global coordinate system O-XYZ. $S^{ij}$ is the flexible body shape function, and ij is the serial number of the discrete unit.

This formulation describes the position vector of any point on the rigid-spring connection member (Equation 7):

$$r_{i}=T\left({\bar{r}}_{i}-{\bar{r}}_{0}\right)+{\bar{r}}_{0}+\sum_{j=1}^{n}N_{j}\left(q_{j}-q_{j}^{0}\right)$$ (7)

To analytically describe the orientation of the moving platform, which simulates the scapula end, the Z-Y-X Euler angle convention is employed. This representation is particularly suitable for the biomimetic design, as the three Euler angles directly correspond to the primary physiological motions of the human lumbar spine: lateral flexion ( α ), anterior-posterior flexion ( β ), and axial rotation ( γ ). The attitude transformation matrix T, which maps vectors from the moving platform’s local coordinate system ( o−xyz ) to the global coordinate system ( O−XYZ ), is therefore defined as a function of these three angles, as shown in Equation 8:

$$T=\left[\begin{array}{ccc} \cos\beta \cos\gamma & \cos\beta \sin\gamma & -\sin\beta \\ \sin\alpha \sin\beta \cos\gamma -\cos\alpha \sin\gamma & \sin\alpha \sin\beta \sin\gamma +\cos\alpha \cos\gamma & \sin\alpha \cos\beta \\ \cos\alpha \sin\beta \cos\gamma +\sin\alpha \sin\gamma & \cos\alpha \sin\beta \sin\gamma -\sin\alpha \cos\gamma & \cos\alpha \cos\beta \end{array}\right]$$ (8)

As the moving platform moves, α, β, and γ vary, causing T to change accordingly, which in turn affects the position vectors $r_{i}$ of points on the rigid-spring member. This rotation matrix is fundamental to the kinematic model, as it establishes a precise mathematical relationship between the motion of the moving platform (the bionic vertebra) and the spatial positions of its connection points α. During rehabilitation motions, the changes in α, β, and γ directly influence the global position vector *r* of any point on the moving platform or the attached rigid-spring member. This is dynamically influenced by T , and is detailed in the subsequent formulation of the position vectors and the moving rod lengths *i* in Equations 6, 9. This dynamic linkage forms the foundation for solving the kinematics of the proposed rigid-flexible coupled mechanism.

The moving rod length $L_{i}$ is related to the motion parameters α, β, and γ of the moving platform. Let the position vector of the connection point on the moving platform under the global coordinate system be $P_{d}$ (α, β, γ), and the position vector of the corresponding point on the fixed platform under the global coordinate system be $P_{d}$ (fixed). Then, the moving rod length can be expressed as Equation 9:

$$L_{i}=\left\|P_{d}\left(\alpha ,\beta ,\gamma \right)-P_{s}\right\|$$ (9)

The deformation $\Delta L_{j}$ of the spring at each discrete cell j is related to the change in the length of the corresponding moving rod $\Delta L_{i}$ , $\Delta L_{j}=k_{j}\Delta L_{i}$ , where $k_{j}$ is a coefficient depending on the spring’s structure and position.

The elastic coordinates $q_{j}$ and the variation of ${q_{j}}_{0}$ are determined by the spring deformation $\Delta L_{i}$ , which is expressed as $q_{j}-q_{j}^{0}=\frac{\Delta L_{j}}{K_{s}}$ in the one-dimensional case if the spring follows Hooke’s law ( $k_{s}$ is the spring stiffness). Substituting Δ $L_{j}$ = $k_{j}$ Δ $L_{i}$ yields $q_{j}-q_{j}^{0}=\frac{k_{j}\Delta L_{j}}{K_{s}}$ .

The velocity vector $r_{i}$ is obtained by taking the first-order derivative of the position vector ${\dot{r}}_{i}$ with respect to time t. According to the derivative rules for complex functions, the velocity vector is given by Equation 10:

$${\dot{r}}_{i}=\frac{dT}{dt}\left({\bar{r}}_{i}-{\bar{r}}_{0}\right)+T\frac{d\left({\bar{r}}_{i}-{\bar{r}}_{0}\right)}{dt}+\frac{d{\bar{r}}_{0}}{dt}+\sum_{j=1}^{n}N_{j}\frac{d\left(q_{j}-q_{j}^{0}\right)}{dt}$$ (10)

Here, $\frac{dT}{dt}$ can be computed for each element in T with respect to the motion parameters α, β, and γ, and then combined with α, β, and γ to obtain $\frac{d\left(q_{j}-q_{j}^{0}\right)}{dt}=\frac{k_{j}}{k_{s}}\frac{d\Delta L_{i}}{dt}$ . Meanwhile, $\frac{d\Delta L_{i}}{dt}$ is obtained by solving for $L_{i}=$ ‖ $P_{d}\left(\alpha ,\beta ,\gamma \right)-P_{s}$ ‖ with respect to time (Equation 11):

$$r_{i}=T\left({\bar{r}}_{i}-{\bar{r}}_{0}\right)+{\bar{r}}_{0}+\sum_{j=1}^{n}N_{j}\frac{k_{j}\Delta L_{i}\left(\alpha ,\beta ,\gamma \right)}{k_{s}}$$ (11)

Here, $\Delta L_{i}\left(\alpha ,\beta ,\gamma \right)=$ ‖ $P_{d}\left(\alpha ,\beta ,\gamma \right)-P_{s}$ ‖ $-L_{i}^{0}$ , with $L_{i}^{0}$ being the initial length of the moving rod (Equations 12, 13):

$${\dot{r}}_{i}=\frac{dT}{dt}\left({\bar{r}}_{i}-{\bar{r}}_{0}\right)+T\frac{d\left({\bar{r}}_{i}-{\bar{r}}_{0}\right)}{dt}+\frac{d{\bar{r}}_{0}}{dt}+\sum_{j=1}^{n}N_{j}\frac{k_{j}}{k_{s}}\frac{d\Delta L_{i}\left(\alpha ,\beta ,\gamma \right)}{dt}$$ (12)

$${\ddot{r}}_{i}=\frac{d^{2}T}{dt^{2}}\left({\bar{r}}_{i}-{\bar{r}}_{0}\right)+2\frac{dT}{dt}\frac{d\left({\bar{r}}_{i}-{\bar{r}}_{0}\right)}{dt}+T\frac{d^{2}\left({\bar{r}}_{i}-{\bar{r}}_{0}\right)}{dt^{2}}+\frac{d^{2}{\bar{r}}_{0}}{dt^{2}}+\sum_{j=1}^{n}N_{j}\frac{k_{j}}{k_{s}}\frac{d^{2}\Delta L_{i}\left(\alpha ,\beta ,\gamma \right)}{dt^{2}}$$ (13)

### 2.3 Static analysis

#### 2.3.1 Analysis of a spring with lateral bending

Springs that are fixed at one end and laterally bent at the other are commonly modeled and analyzed using beam theory in rigid-flexible coupled exoskeletons. This approach is particularly relevant under physiological motion loads that induce large deflection and coupled deformations (Li et al., 2019). Such springs act as bionic analogs to the lumbar vertebral-disc-ligament complex during human spine-like motions, making this analytical framework highly relevant to the current study.

1. The spring undergoes elastic deformation within the material’s yield limit, with axial deformation negligible compared to its original length. Therefore, the spring’s overall length and cross-sectional radius are considered constant during motion, ensuring the stability in its supportive and load-bearing functions.
2. The self-weight of the spring is significantly smaller than the external physiological loads (50–120 N, corresponding to human muscle forces during lumbar motion). Consequently, the deflection caused by self-weight is neglected, which is consistent with common assumptions in rigid-flexible coupled exoskeleton dynamics analysis.
3. The spring does not experience plastic deformation under the designed driving loads, as the maximum stress remains far below the yield strength of structural steel. This ensures repeatability and safety during rehabilitation training.

Under the action of two mutually perpendicular lateral forces, $F_{x}$ and $F_{y}$ , and a force couple $M_{0}$ , the spring undergoes a large lateral deflection. For small deflections, the curvature can be approximated as $k\approx \frac{d^{2}v}{dx^{2}}$ . However, for large deflections, such as the maximum forward bending angle of the human spine, which reaches 60°, the nonlinear terms of the deflection derivative cannot be ignored. The accurate curvature of the spring’s deflection curve is defined as kx=d2v/dx21+dv/dx232 , for any cross-section of the spring. At any cross-section of the spring, the bending moment is related to the curvature by the equation $M\left(x\right)=EI\cdot k\left(s\right)$ . Under large deflection conditions, this nonlinear curvature must be considered to accurately describe the spring’s behavior. Finally, the torsion spring’s center diameter is a key geometric parameter influencing bending stiffness and curvature (Equation 14):

Mx=EI·d2v/dx21+dv/dx232 (14)

Here, x is the coordinate along the axial direction of the spring, $v\left(x\right)$ represents the lateral deflection of the spring at position x , $k\left(x\right)$ denotes the curvature of the spring deflection curve at x , $M\left(x\right)$ is the bending moment of the spring at position x , EI is the bending stiffness of the spring, where E is the elastic modulus of the spring material, I is the moment of inertia of the spring wire, and d is the diameter of the spring wire.

To derive the specific expression of $M\left(x\right)$ , take the fixed end of the spring as the origin O of the global coordinate system X−XYZ . Let the x-axis align with the original axis of the spring, pointing toward the free end. At any cross-section located at position x , the bending moment consists of three components: The moment caused by the horizontal lateral force $F_{y}$ , the moment caused by the vertical lateral force $F_{x}$ , and the applied force couple $M_{0}$ . The direction of the bending moment follows the right-hand rule and is consistent with the positive direction of curvature. Therefore, the bending moment at position x is expressed as Equation 15:

$$M\left(x\right)=F_{y}\left(L-x\right)-F_{x}v\left(x\right)+M_{0}$$ (15)

Here, $F_{x}$ is the horizontal lateral force applied at the free end of the spring, and $F_{y}$ is the vertical lateral force applied at the free end of the spring. The torque exerted by $M_{0}$ at the free end causes the spring to rotate within the plane perpendicular to the x-axis. L represents the original (undeformed) length of the spring.

Substituting this expression of *M(x)* into Equation 14 and differentiating with respect to time yields (Equation 16):

$$\frac{dM\left(x\right)}{dx}=-F_{y}-F_{x}\left(\frac{dv}{dx}\right)$$ (16)

By combining Equations 14, 15 and eliminating $M\left(x\right)$ , the lateral bending equation for large deflections is obtained (Equation 17):

d2v/dx21+dv/dx232=1EIFyL−x−Fxvx+M0 (17)

Let $\frac{dv}{dx}=\tan\theta$ , then $\frac{d^{2}v}{dx^{2}}=sec^{2}\theta \cdot \frac{d\theta }{dx}$ . Substitute this into Equation 17, apply 1+tan2⁡θ32=sec3θ , and rearrange to obtain Equation 18:

$$\frac{d\theta }{dx}=\frac{1}{EI}\left[F_{y}\left(L-x\right)-F_{x}v\left(x\right)+M_{0}\right]\cos^{3}\theta$$ (18)

Here, θ is the tangential angle of the spring at position *x*.

The geometric relationship between the axial deformation $u\left(x\right)$ and the lateral deflection of the spring is given by $\frac{du}{dx}=1-\sqrt{1+{\left(dv/dx\right)}^{2}}$ . Combining this relationship with Equation 18 establishes the coupling Equation 19:

$$\left\{\begin{array}{l} \frac{d\theta }{dx}=\frac{1}{EI}\left[F_{y}\left(L-x\right)-F_{x}v\left(x\right)+M_{0}\right]\cos^{3}\theta \\ \frac{du}{dx}=1-\sec\theta \end{array}\right.$$ (19)

Substituting Equation 19 into Equation 18 gives the final form Equation 20:

$$\frac{d\theta }{dx}=\frac{1}{EI}\left[F_{y}\left(L-x\right)-F_{x}\int_{0}^{x}\tan\theta d\tau +M_{0}\right]\cdot \cos^{3}\theta$$ (20)

Here, $v\left(x\right)=\int_{0}^{x}\tan\theta d\tau$ $ds=\sqrt{{\left(dx\right)}^{2}+{\left(dv\right)}^{2}}=dx\cdot \sec\theta$ is the differential arc length element, and the integral is taken over the interval $x\in \left[0,L\right]$ . The tangent angle satisfies the boundary conditions at the fixed and free ends of the spring ( θ=0 at x=0 ), thus Equation 21:

$$s=\int_{0}^{L}\sec\theta \left(x\right)dx\;\theta \left(L\right)=\int_{0}^{L}\frac{1}{EI}\left[F_{y}\left(L-x\right)-F_{x}v\left(x\right)+M_{0}\right]\cdot \cos^{3}\theta \left(x\right)dx$$ (21)

The total length of the spring, $L_{total}=L+u\left(L\right)$ , after accounting for axial deformation $u\left(L\right)=\int_{0}^{L}\left(1-\sec\theta \right)dx$ , is expressed as Equation 22:

$$L_{total}=L+\int_{0}^{L}\left[1-\sqrt{1+{\left(\frac{dv}{dx}\right)}^{2}}\right]dx$$ (22)

According to the spring configuration, $\cos\theta =\frac{dx_{s}}{d\tau }$ and $\sin\theta =\frac{dy_{s}}{d\tau }$ from Equation 20 can be determined along the centerline of the spring at any point with coordinates $\left(x\left(s\right),y\left(s\right)\right)$ (Equations 23, 24):

$$x\left(s\right)=\int_{0}^{s}\cos\theta \left(\tau \right)d\tau$$ (23)

$$y\left(s\right)=\int_{0}^{s}\sin\theta \left(\tau \right)d\tau$$ (24)

From the perspective of static analysis, in addition to examining the lateral bending and torsion of the spring, the overall force balance of the mechanism must also be considered. When the mechanism is in static equilibrium, the external forces and moments acting on the moving platform must be balanced by the spring forces and resulting deformations.

Let $\vec{F}=\left(F_{x},F_{y,}F_{z}\right)$ denote the external force acting on the moving platform, and $\vec{M}=\left(M_{x},M_{y,}M_{z}\right)$ the external moment. Let ${\vec{F}}_{s1},{\vec{F}}_{s2},{\vec{F}}_{s3}$ represent the spring force acting on the moving platform, corresponding to the forces from each of the three springs. Applying the force and moment balance equations yields (Equations 25, 26):

$$\sum_{i=1}^{3}\vec{F_{i}}=\left({\vec{F}}_{ext}\right)$$ (25)

$$\sum_{i=1}^{3}\vec{r}A_{i}\times \vec{F_{i}}={\vec{M}}_{ext}$$ (26)

Here, ${\vec{r}}_{Ai}$ is the position vector from the point $A_{i}$ on the spring to the center of mass of the moving platform.

For the spring force ${\vec{F}}_{si}$ , according to Hooke’s law, its magnitude is proportional to the deformation of the spring within its elastic limit. For a given spring ***i***, let the stiffness coefficient be $k_{i}$ and the deformation be $\Delta L_{i}$ . Then, the magnitude of the spring’s elastic force is $F_{si}$ = $k_{i}$ Δ $L_{i}$ .

The deformation $\Delta L_{i}$ of the spring can be calculated from the change in length of the moving rod $L_{i}$ and the initial length $L_{0i}$ of the spring as Δ $L_{i}$ = $L_{i}$ - $L_{0i}$ .

A complete model of the mechanism’s statics is obtained by substituting the expression for the spring elasticity into the force and moment equilibrium Equations 27, 28:

$$\sum_{i=1}^{3}k_{i}\left(l_{i}-l_{i0}\right)\vec{n_{i}}=\left({\vec{F}}_{ext}\right)$$ (27)

$$\sum_{i=1}^{3}\vec{r}A_{i}\times k_{i}\left(l_{i}-l_{i0}\right){\vec{n}}_{i}={\vec{M}}_{ext}$$ (28)

Here, $\vec{n_{i}}$ is the unit vector in the elastic direction of spring *i*, and $k_{i}$ is the stiffness coefficient of the i -th spring.

Using the static model described above, the deformation and force of each spring can be determined for known external forces and moments. This provides a critical basis for the structural design and strength calibration of the mechanism. For instance, during the design process, the required spring stiffness coefficients and initial lengths can be calculated using this model based on the expected external loads. This ensures that the mechanism maintains stable performance under a range of operating conditions.

#### 2.3.2 Spring torsion analysis

The maximum forward bending angle of the human spine is 35°, which represents a relatively large range of motion. Under this condition, torsion springs are primarily subjected to torque. Due to the very small helix angle during torsion, it can be approximated as a constant value, α_0_. Thus, at any cross-section of the spring under torsion, only the bending moment M = T acts. When a torque T is applied at both ends of the spring, the torsion angle φ is calculated as: $\phi =\frac{TL}{GI_{P}}$ . For a circular cross-section spring wire with $I_{P}=\frac{\pi d^{4}}{32}$ , the torsion angle ϕ of the spring is calculated as Equation 29:

$$\phi =\frac{32TL}{G\pi d^{4}}$$ (29)

Here, d is the torsion spring’s center diameter (mm), T is the torque applied to the torsion spring, G is the shear modulus of the spring material, $I_{P}$ is the polar moment of inertia of the spring wire, and L is the original (undeformed) length of the spring.

As the human spine rotates, the effective spring length increases. Therefore, the rotational deformation must include both the torsion caused by the applied torque and the additional deformation due to the rotation angle. This rotational deformation is expressed as Equation 30:

$$\Delta \phi =\frac{T\left(l_{1}^{2}+l_{2}^{2}\right)}{2EI}$$ (30)

In the above equation, $l_{1}$ and $l_{2}$ denote the lengths of the two outer torsion arms of the spring. The bending stiffness of the spring is given by EI , where E is the elastic modulus and I is the moment of inertia of the spring wire cross-section.

The deformation energy of the torsion spring is calculated as Equation 31:

$$U=\frac{T^{2}L}{2GI_{P}}$$ (31)

The maximum stress in the inner coil of the torsion spring under torque is evaluated as Equation 32:

$$\tau_{\max}=\frac{16T}{\pi d^{3}}$$ (32)

The torsional stiffness of the torsion spring is expressed as Equation 33:

$$k_{T}=\frac{G\pi d^{4}}{32L}$$ (33)

When the torsion spring is twisted in the direction opposite to its normal rotation, the curvature coefficient *K*_0_ is given by Equation 34:

$$K_{0}=\frac{4C-1}{4C-4}+\frac{0.615}{C}$$ (34)

Here, the rotation ratio is expressed as C = D/d, with D being the center diameter of the torsion spring and *d* being the wire diameter.

### 2.4 Simulation analysis

#### 2.4.1 Kinematic simulation

In this analysis, the range of lumbar motion required for common daily activities is considered as $\alpha \subset \left(0,\pi /9\right)$ , $\beta \subset \left(0,\pi /3\right)$ , γ⊂0,π6 . In Adams, both kinematic and static simulations were performed on a flexible parallel mechanism representing the lumbar joint. To verify the mechanism’s performance during motion, simulations were performed for lateral bending, flexion-extension, and axial rotation movements.

The lateral flexion mechanism allows for left and right lateral bending through the movement of the moving rods on both sides. This behavior was validated by observing the motion of the moving rods in the simulation.

#### 2.4.2 Kinetic analysis

The lumbar abdominal muscles are the main source of force for human waist movements. Their role is not only to provide support for the body but also to offer protection and ensure the therapeutic effectiveness of rehabilitation devices. These muscles help prevent injury during treatment and controlled movement. The magnitude of lumbar muscle forces is influenced by factors such as gender, age, muscle mass, and training level. The force also varies depending on the joint angle at which it acts. Explosive actions, such as throwing, generate higher instantaneous forces, while static or slow movements produce lower forces. As a rehabilitation device, only forces relevant to controlled, daily movements are considered; high-impact or explosive forces are excluded. For example, during forward flexion, the anterior deltoid fascicle generates forces of approximately 50–120 N. During lateral flexion, the middle deltoid fascicle generates forces of approximately 30–80 N. During upright posture, the lumbar muscles generate forces of approximately 20–50 N. By analyzing the body’s force-generating points and establishing structural models for finite element simulation, it is shown in Figure 9 that the forces generated by the model are consistent with the physiological range of lumbar muscle forces, approximately 20–50 N.

### 2.5 Human experiment

#### 2.5.1 Introduction of experimental device

**FIGURE 5:**
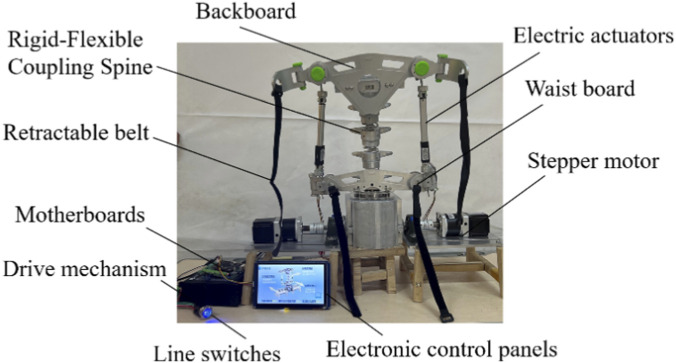
Main components of the experimental setup.

The rigid-flexible coupling spine rehabilitation mechanism was first modeled and analyzed through simulation. Following the simulation, a physical test machine was constructed. The experimental device is primarily composed of a stepper motor, a back plate, an electric actuator, a drive device, and other auxiliary components. The overall structure of the device is shown in Figure 5.

#### 2.5.2 Experimental design

##### 2.5.2.1 Forward flexion test

Volunteers were guided to perform a forward bending movement. Due to the limited angle of human spinal back-flexion, this experiment only verified the feasibility of the device’s back-flexion movement. No detailed data measurement or recording was performed for this movement. During the test, volunteers were instructed to bend forward slowly and hold the position for 5 s upon reaching the maximum range of motion. Performing the movement slowly improves measurement accuracy and reduces errors caused by rapid motion. Holding the position for 5 s allows the measuring instrument to fully capture steady-state data, enhancing the validity of the measurements. The experimental setup for this test is shown in Figure 10a.

The angle change of the waist and back was recorded, and the experimental data were compared with simulation results. The resulting comparison of angle changes is shown in Figure 10b.

##### 2.5.2.2 Lateral flexion test

In this test, volunteers were guided to perform lateral flexion movements. During the test, each volunteer performed the lateral flexion slowly according to the instructions and held the position for 5 s upon reaching the maximum range of motion. Performing the movement slowly allows the measuring instruments to record the motion data more accurately and reduces errors caused by higher speeds. Holding the movement for 5 s ensures that the steady-state movement parameters are captured, improving the reliability of the data. The experimental procedure for the lateral flexion exercise is shown in Figure 11a.

The built-in measuring instruments of the rehabilitation device were used to record angular changes of the waist and back during the motion. These experimental data were then compared with the simulation results. The comparison of angular changes between the experimental and simulation data is shown in Figure 11b.

##### 2.5.2.3 Rotary motion test

In this test, volunteers were guided to perform rotational (axial) movements of the spine. The experimental procedure for the rotary motion testis shown in Figure 12a.

During the test, waist and back angle changes were recorded using the device’s built-in measuring instruments. These experimental measurements were then compared with the simulation data. The resulting comparison of angular changes is shown in Figure 12b.

### 2.6 Sample size calculation for volunteers

The sample size was calculated using the precision estimation formula for continuous measurement data. Two key performance indicators were used as primary outcomes: lumbar motion angle (°) and maximum motion speed (°s) . These indicators are critical for verifying the mechanism’s kinematic compatibility with the human body. The formula for calculating sample size is Equation 35:

$$n={\left(\frac{Z_{\alpha /2}\times \sigma }{\delta }\right)}^{2}$$ (35)

Here, $Z_{\alpha /2}$ is the critical value of the standard normal distribution ( α = 0.05, two-sided test, Z=0.0521.96 , σ is the standard deviation of the outcome indicator from a preliminary small-sample pre-test, δ is the allowable measurement error, set to 10% of the pre-test indicator mean (the minimum clinically acceptable error for rehabilitation robot performance testing).

A pre-test was conducted on three healthy volunteers, consistent with the inclusion criteria for the formal experiment. The pre-test results yielded the following key indicators:

Average lumbar forward flexion angle of 38.2° ( σ=2.1° ) (Equation 36):

$$n={\left(\frac{1.96\times 2.1}{38.2\times 10\%}\right)}^{2}\approx 1.4$$ (36)

Average maximum motion speed: 15.1°/s ( σ=0.8°s ) (Equation 37):

$$n={\left(\frac{1.96\times 0.8}{15.1\times 10\%}\right)}^{2}\approx 1.1$$ (37)

The theoretical minimum sample size calculated from these pre-test results was 2 participants.

To account for individual differences in lumbar motion, potential measurement errors, and the need for statistical validity in human-machine experiments, the sample size was expanded by a factor of 5, a conventional practice in rehabilitation robot prototype testing. This expansion resulted in a final sample size of 10 participants. This approach ensures that the experimental results are representative and reliable, even when minor variations in volunteer performance or measurement noise are present. Moreover, volunteers with spinal disorders were excluded to prevent interference from pathological factors.

## 3 Results

### 3.1 Simulation results

#### 3.1.1 Lateral flexion simulation

**FIGURE 6:**
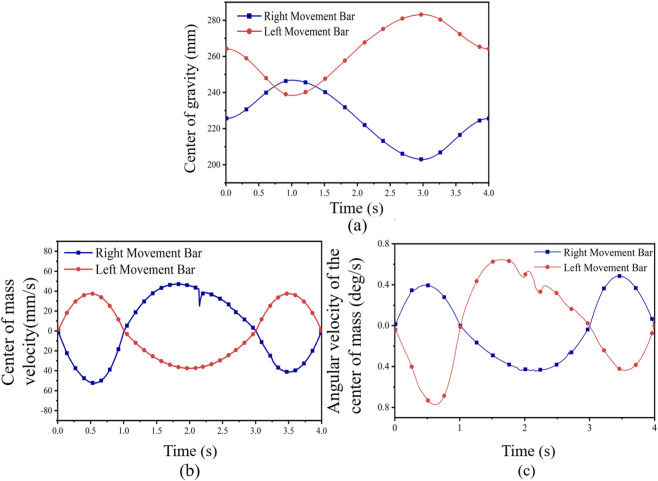
Simulation curves of the center-of-mass motion during lateral flexion of the moving rods. **(a)** center of gravity. **(b)** center of mass velocity. **(c)** angular velocity of the center of mass.

As shown in Figure 6a, the initial positions of the right and left kinematic bars were 225.60 mm and 264.22 mm, respectively. Over a 4-s cycle, their positions changed alternately. The right bar increased while the left decreased, and then the motion reversed. By the end of the cycle, their positions returned to approximately 225.61 mm and 264.22 mm, completing a full periodic alternating movement. It is noted here that this motion aligns with the biomechanical coordination of the human waist.

As shown in Figure 6b, the left connecting rod initially moved right at approximately 36.98 mm/s, while the right rod moved left at about −52.34 mm/s. During the peak period, their speeds reached maximum values of 45.32 mm/s (left) and −37.52 mm/s (right). This simulates the dominant alternating pullback and advancement of the waist during walking or arm swinging.

Moreover, Figure 6c shows that the left connecting rod’s angular velocity first decreased from 0 to −0.76 deg/s, indicating backward acceleration. Meanwhile, the right rod’s angular velocity increased to a peak of 0.39 deg/s, indicating forward acceleration. Subsequently, both angular velocities alternated between positive and negative values, reflecting multiple oscillations and directional reversals. These oscillations gradually decayed and stabilized, consistent with the dynamic characteristics of human body coordination.

#### 3.1.2 Spinal segment coordination

**FIGURE 7:**
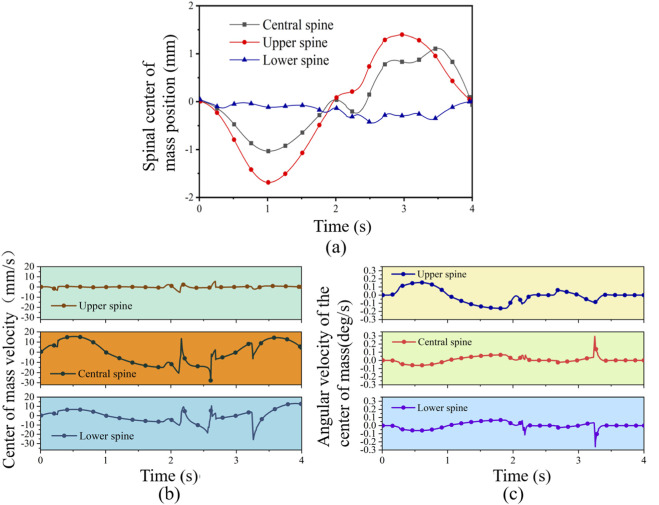
Simulation curves of spinal lateral flexion motion. **(a)** center of mass position curve. **(b)** center of mass velocity curve. **(c)** center of mass angular velocity curve.

As shown in Figure 7a, during the initial stage, the lower spine moved from 0 mm in the negative direction to approximately −0.12 mm. It then rapidly shifted to the positive direction, attaining a peak of 0.02 mm at 0.65 s. Overall, the lower spine’s movement was relatively smooth, oscillating around the horizontal axis. The middle spine initially moved rapidly in the negative direction, reaching −1.03 mm at 1.02 s, before reversing and rising to −0.24 mm at 2.3 s. Its speed accelerated during this phase, followed by small fluctuations, and eventually settled at −0.07 mm, close to the initial position. The middle spine exhibited a greater range of motion than the lower spine. Additionally, the upper spine moved slightly in the negative direction from 0 mm to −1.69 mm at 1.01 s, then shifted positively to 1.40 mm at 2.97 s, and finally returned to −0.00145 mm. The spinal trajectory demonstrates clear segmental coordination: the lower spine dominates backward and forward movement, the middle spine adjusts with moderate amplitude to maintain balance, and the upper spine exhibits the largest amplitude with more intense dynamics. It is noted that the motion pattern is consistent with the “wave-like” mechanical transmission characteristics of the human spine.

As shown in Figure 7b, the center of mass velocity of the lower spine initially increased from 0 mm/s to 6.7 mm/s. It then gradually decreased to a minimum of −6.51 mm/s at 1.87 s, followed by a fluctuating rise to a maximum of 13.1 mm/s at 3.9 s. The middle spine showed a similar undulating velocity pattern, with a maximum of 15.49 mm/s at 0.52 s and a minimum of −20.60 mm/s at 2.12 s. The upper spine moved relatively smoothly, showing a near-linear trend in velocity. It reached a minimum of −5.39 mm/s at 2.14 s and a maximum of 5.66 mm/s at 2.68 s. Overall, these velocity patterns align with the natural dynamics of human lateral spine flexion.

Figure 7c shows that the angular velocity of the lower spine’s center of mass initially decreased from 0 deg/s to −0.06 deg/s. It then gradually increased, reaching a maximum of 0.07 deg/s at 1.79 s, followed by fluctuations and a minimum of −0.26 deg/s at 3.25 s. The angular velocity of the middle spine followed a similar trend but with peak values opposite to those of the lower spine, reaching a maximum of 0.04 deg/s in 3.23 s. The upper spine showed wavelike changes in angular velocity, peaking at 0.15 deg/s at 0.6 s. Overall, the angular velocity profiles of the spine segments are consistent with the lateral flexion dynamics of the human body.

#### 3.1.3 Rotational motion simulation

**FIGURE 8:**
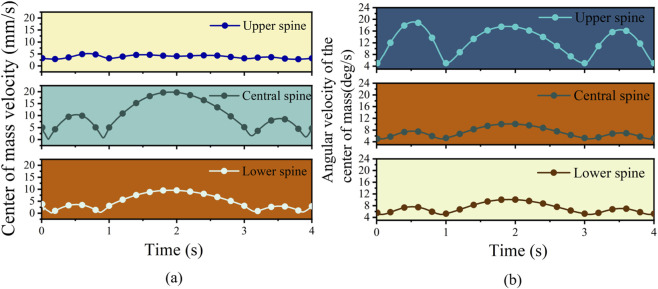
Simulation curves of spinal rotation motion. **(a)** center of mass velocity curve. **(b)** center of mass angular velocity curve.

As shown in Figure 8a, the velocity of the lower spine initially fluctuated rapidly from 1.27 mm/s, reaching a peak of 3.97 mm/s at 0.02 s, followed by a rapid decrease to 0.36 mm/s. During the middle stage of spinal motion, the lower spine’s velocity gradually increased, reaching 3.10 mm/s at 1 s, and then reached a maximum of 9.54 mm/s at 1.87 s. This indicates that the lower spine continued to accelerate its rotation during the middle phase of movement. The middle spine exhibited a trajectory similar to that of the lower spine, with its rotational center-of-mass velocity slightly higher, reaching a maximum of 19.80 mm/s. The upper spine moved smoothly, with overall velocities ranging between 0.2 mm/s and 0.9 mm/s.

Moreover, Figure 8b shows that during the initial movement phase, the lower spine exhibited a slight zigzag pattern, although its overall rotation remained relatively smooth. Its maximum angular velocity reached 10.11 deg/s at 1.9 s. The middle spine’s rotational behavior and angular velocity trends were generally similar to those of the lower spine. In contrast, the upper spine showed larger fluctuations in angular velocity, yet its rotation was also smooth overall, peaking at 19.06 deg/s. The variation patterns of all three spinal segments are consistent with the typical rotational dynamics of the human lumbar region.

#### 3.1.4 Structural deformation analysis

**FIGURE 9:**
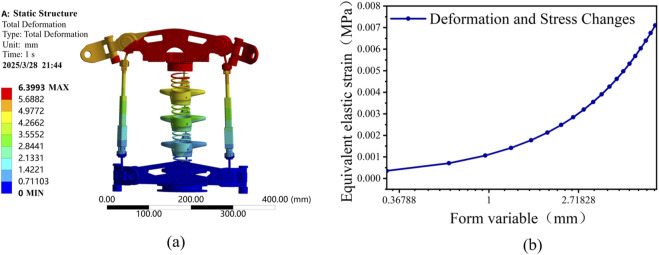
Deformation and elastic stress-strain curve of lumbar spine structure. **(a)** lumber deformation. **(b)** elastic strain contour plot o the lumbar spine.

As shown in Figure 9a, with structural steel used as the structural material, the maximum deformation of the rehabilitation mechanism reached approximately 6.40 mm when force was applied by the human body. This deformation is consistent with the design expectations. If the deformation were too small, excessive force from the human body could cause discomfort or harm. Conversely, excessive deformation could reduce the rehabilitation effect due to significant bending and insufficient support during use.

Additionally, Figure 9b depicts the measured deformation versus elastic strain graph, indicating that deformation gradually increases with elastic stress. When the stress reaches its maximum value, the deformation is 6.40 mm, accompanied by a moderate degree of bending.

### 3.2 Experimental results

#### 3.2.1 Forward flexion test

**FIGURE 10:**
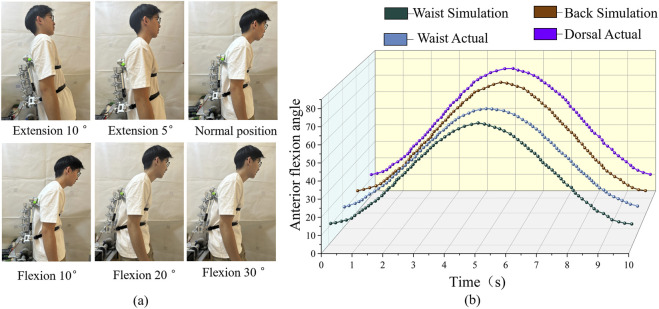
Flexion-extension trajectory curve. **(a)** flexion and extension experiment. **(b)** flexion-extension trajectory curve.

As shown in Figure 10b, the changes in the angle between the lumbar region and the back were recorded by the built-in measuring instruments of the rehabilitation device while volunteers performed forward bending exercises. These measurements were then compared with the simulation data obtained previously. The results show that the actual maximum lumbar forward flexion angle was 68.6°, while the maximum back rotation angle was 77.5°. Both values fall within the typical range of maximum human rotational movement. The simulation results demonstrate a high degree of consistency with the actual measurements. The back exhibited a greater angular change (approximately 8.9°) than the lumbar region, reflecting the synergistic kinematic relationship between the back and lumbar spine during forward flexion. Overall, the motion pattern closely replicates natural human biomechanics. These findings indicate that the rehabilitation device can effectively simulate real human motion during forward bending. The achieved angular profiles meet the design expectations, validating the device’s efficacy for forward flexion rehabilitation training. Moreover, the results confirm the rationality of the rigid-flexible coupling design architecture for this specific movement mode.

#### 3.2.2 Lateral flexion

For the lateral flexion, the results showed that the maximum lumbar lateral flexion angle was 60.4°, while the maximum dorsal lateral flexion angle was 69.1°. Both values fall within the typical range of lateral flexion in human movement.

**FIGURE 11:**
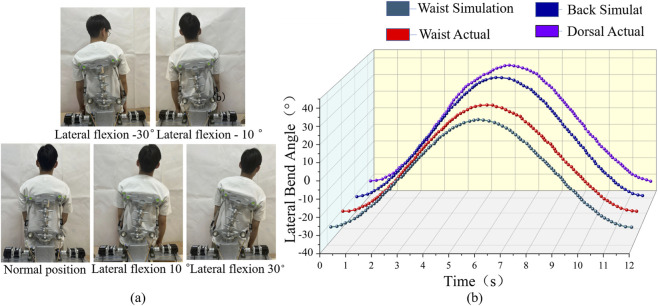
Lateral flexion trajectory curve. **(a)** Lateral flexion movement experiment. **(b)** spinal Lateral flexion trajectory curve.

Comparison between the simulation and actual measurements of lumbar lateral flexion demonstrated a high degree of agreement. It is noted here that the back exhibited greater angular change than the lumbar region, by approximately 8.7°, which aligns with the actual biomechanics of the human body during lateral flexion. These findings indicate that the rehabilitation device can accurately simulate human movement during lateral flexion. They also confirm that the device’s design is reasonable and effective for lateral flexion functionality (Figure 11b).

#### 3.2.3 Rotation

For rotational motion, the results showed that the maximum lumbar rotational angle was 58.52°, while the maximum back rotational angle reached 67.58°. These values are consistent with the typical range of human rotational motion.

**FIGURE 12:**
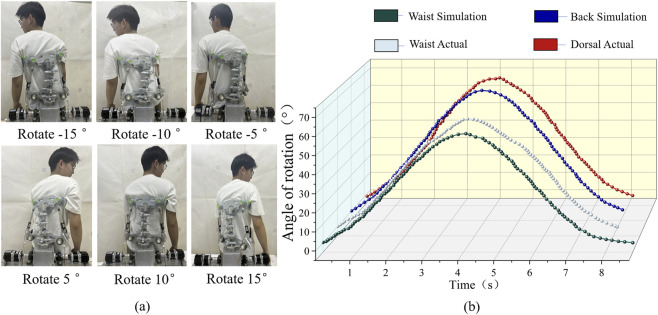
Axial rotation trajectory curve. **(a)** rotary motion experiment. **(b)** lumber rotation motion trajectory curve.

It is worth noting here that the simulation results for lumbar rotation closely matched the actual measurements. The back exhibited a larger angular change than the lumbar region, by approximately 9.0°, consistent with natural human rotational mechanics. These results fully verify the performance of the rehabilitation device in rotational motion. They demonstrate that the device’s design supports human rotational rehabilitation training with high accuracy and reliability (Figure 12b).

### 3.3 Human experimental results

In this investigation, the participant group consisted of ten volunteers, including 7 males and 3 females, with a mean age of 19.8 ± 1.5 years (range: 18–22 years). The mean height and weight of the participants were 174.6 ± 7.4 cm and 66.9 ± 8.6 kg, respectively. All subjects reported no relevant medical history.

**FIGURE 13:**
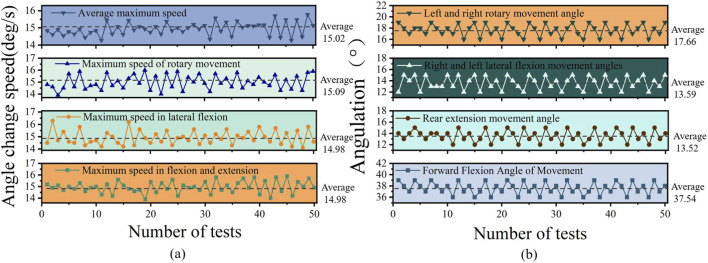
Data analysis chart. **(a)** angle change speed curve. **(b)** angulation curve.

Each volunteer performed five sets of experiments, during which movement angles and speeds were recorded. The average values across the volunteers were calculated and are presented in Figure 13a.

The results indicate that the average angles achieved were: 37.6° for forward flexion, 13.56° for backward flexion, 13.62° for left/right lateral flexion, and 17.5° for left/right rotation. All measured values were close to or within the designed operational ranges: 0°–40° for forward flexion, 0°–15° for backward flexion, 0°–15° for lateral flexion, and 0°–20° for rotation. Although individual variations were observed, the overall motion angles fell within a reasonable span. This demonstrates that the mechanical structure of the rehabilitation device can effectively support diverse lumbar motions and satisfy fundamental rehabilitation training requirements (Figure 13b).

Similarly, the average maximum movement speed across all volunteers was 14.94°/s, closely matching the design target of 14.90°/s (Figure 13b). Flexion-extension movements showed slightly higher speeds than lateral flexion and rotation, likely due to greater daily familiarity and proficiency with these motions. However, the differences were minor, indicating that the device provides balanced actuation across all motion directions. These results confirm that the device’s power system and control algorithm can reliably drive lumbar movements, meeting the basic speed and performance requirements for rehabilitation training.

## 4 Discussion

The core objective of this study was to address the limitations of existing back rehabilitation mechanisms, including fixed motion axes, poor joint alignment, and inadequate flexibility. To overcome these limitations, a rigid-flexible coupled parallel rehabilitation mechanism supported by compression springs was proposed. The integration of rigid driving components with flexible bionic structures retains the precision and stability of parallel mechanisms while also mimicking the viscoelastic properties of the human lumbar vertebra-disc-ligament complex. This design achieves a balance between precise motion control and biomechanical compatibility. The simulation and experimental results collectively validate the rationality of the proposed mechanism. However, further analysis of its performance, implications, and experimental design rationale is necessary to fully contextualize its value in the field of rehabilitation robotics.

This research makes a substantial contribution to both the theoretical foundations and practical applications of spinal rehabilitation robotics. From a theoretical standpoint, the study develops a comprehensive kinematic and static mathematical model for rigid-flexible coupled parallel mechanisms used in lumbar rehabilitation. The model incorporates the numerical solution of large-deflection bending and torsional deformation of springs under physiological loads. The derivation of calculation methods for the spring’s motion-end bending angle and torsion angle enhances the mechanical analysis framework for flexible components in rehabilitation robots. The proposed modeling framework is generalizable to other parallel mechanisms with flexible supports, offering a unified theoretical basis for the development of rehabilitation robots targeting limbs, joints, and the spine. This work addresses a critical research gap by enabling the design of simple yet effective flexible spine analogs within parallel structures specifically tailored for back rehabilitation.

From a practical application perspective, the proposed rigid-flexible coupled lumbar rehabilitation mechanism effectively addresses key limitations of traditional rigid exoskeletons, including joint misalignment, restricted motion range, and poor wearing comfort. Its three-degree-of-freedom (3-DoF) motion capability, covering flexion/extension, lateral flexion, and rotation, closely replicates the physiological motion space of the human lumbar spine. The adjustable shoulder-lumbar fixation device accommodates individual differences in body size and injury conditions, overcoming common drawbacks of existing wearable exoskeletons, such as difficult donning and doffing and excessive body load. Under a 120 N anterior deltoid fascicle force, the mechanism achieves a maximum deformation of 6.40 mm, providing effective load buffering. This reduces the risk of secondary lumbar injury during rehabilitation while maintaining structural support for functional training. The device offers a novel technical solution for both clinical lumbar rehabilitation and home-based exercise for patients with lumbar muscle strain, lumbar disc herniation, and mild lumbar degenerative diseases. It holds significant practical value in enhancing rehabilitation outcomes for patients and in addressing the increasing incidence of lumbar disorders among younger populations caused by poor working and living postures.

Additionally, this study broadens the application of rigid-flexible coupling technology within spinal rehabilitation robotics. In contrast to existing rigid-flexible coupled rehabilitation devices, which primarily target limb joints such as the ankles, knees, and upper limbs, this research innovatively applies rigid-flexible coupling structures to the lumbar spine. This is a region characterized by complex motion patterns and high structural support demands. This approach opens a new research direction for the miniaturization, intelligent control, and bionic design of spinal rehabilitation robots. It also fosters cross-disciplinary integration between mechanical engineering and rehabilitation medicine, advancing both fields simultaneously.

A key finding of this study is the mechanism’s ability to replicate the natural kinematics of the human spine across three degrees of freedom. The maximum motion angles, 68.6° for lumbar forward flexion, 60.4° for lateral flexion, and 58.5° for rotation, align closely with the physiological motion range of healthy adults (Heo et al., 2020; Zuo et al., 2014), highlighting its potential to support functional rehabilitation training. Notably, the average maximum movement speed of 14.94°/s nearly matches the design target of 14.90°/s. The balanced speed performance across different motion directions indicates that the drive system and control algorithm effectively mitigate excessive mechanical impacts, a key advantage over traditional rigid exoskeletons, which often generate abrupt forces during motion (Guo et al., 2020; Li et al., 2023). Furthermore, the mechanism’s maximum deformation of 6.40 mm under a 120 N anterior deltoid fascicle force demonstrates its capacity to buffer external loads. This load-buffering ability reduces the risk of secondary lumbar injury while maintaining structural support for effective rehabilitation training.

Compared with existing rehabilitation technologies, the proposed mechanism introduces distinct innovations in both structural design and biomechanical mimicry. Unlike rigid parallel lumbar rehabilitation robots (e.g., Guo et al., 2020), which are prone to joint misalignment due to fixed axes, the compression spring-supported rigid-flexible structure adaptively aligns with the dynamic motion axes of the human spine. This design significantly improves wearing comfort and motion compatibility. In comparison to flexible rehabilitation devices that use bellows-type actuators (Tian et al., 2023) or elastic elements (Kim et al., 2021), the parallel mobile rod drive offers higher motion precision and controllability, effectively addressing the common trade-off between flexibility and stability. Furthermore, the adjustable shoulder fixation device accommodates individual differences in body size and injury conditions, overcoming limitations of wearable exoskeletons that often suffer from inconvenient donning/doffing or excessive weight bearing (Al-Dahiree et al., 2022; Li et al., 2023). This combination of bionic design and engineering practicality fills a critical gap in current back rehabilitation mechanisms. It provides a new paradigm for achieving both biomechanical adaptability and effective functional training.

Despite the contributions and innovative highlights, this study shows some notable limitations that constrain the generalizability of its findings and the immediate applicability of the proposed rigid-flexible coupling back rehabilitation mechanism. First, all kinematic and functional validations were conducted exclusively on healthy young adults with no history of impairment or musculoskeletal disorders. This homogeneity of samples fails to capture the diversity of physiological conditions, movement constraints, or pain profiles that are characteristic of the target clinical population, including individuals with lumbar degenerative diseases (such as lumbar disc herniation, chronic lumbar muscle strain, and spinal stenosis). Consequently, critical performance metrics, including wearing comfort for patients with structurally compromised lumbar regions, long-term adherence to rehabilitation training among patients with varying symptom severity, and the actual therapeutic efficacy in restoring motor function and alleviating chronic low back pain, remain untested and unconfirmed by rigorous clinical trials. This gap not only constrains the immediate application of the proposed solution to real-world rehabilitation practice but also leaves open the possibility of adverse effects of the device, such as secondary pressure injury or excessive spinal stress, when it is used by patients with compromised lumbar structural integrity. Second, the proposed robot relies on a control system with predefined trajectory tracking, which runs on a fixed set of motion parameters for all users without dynamic adjustment and lacks adaptive impedance control informed by real-time physiological and mechanical feedback—such as surface electromyography signals reflecting lumbar muscle activation or fatigue, and human-machine interaction forces. This lack of adaptive control precludes personalized training of the device and makes it impossible to tailor the motion parameters, such as intensity, speed, and range of its motions, to the individual’s muscular capacity, rehabilitation progress, or instantaneous physical responses. Such an one-size-fits-all approach runs counter to the core principle of personalized rehabilitation in clinical practice. Another limitation stems from the prototype’s material composition: its extensive use of structural steel, though having ensured structural rigidity and load-bearing capacity, results in a relatively high overall mass. This substantially undermines the device’s portability, making the device ill-suited for home-based rehabilitation, which is critical for long-term lumbar disease recovery; Moreover, the additional load introduced by the device onto the user’s trunk and lumbar regions may cause muscle fatigue and discourage regular use, particularly among the older users or those with pre-existing lumbar muscle weakness, for whom the extra mass may offset some therapeutic benefits of the device. Consequently, the device’s adoption in community and home-based rehabilitation settings is likely to be further limited.

## 5 Conclusion

This study proposed a rigid-flexible coupled parallel back rehabilitation mechanism that integrates a rigid electric actuator with a flexible bionic spine. The mechanism enables a wide range of bending, lateral flexion, and rotational motions while effectively addressing joint misalignment issues commonly observed in traditional rigid devices. A biomechanically informed mathematical model was established, and both simulation and experimental tests confirmed that the mechanism’s kinematic performance closely aligns with the designed operational ranges and natural human movement patterns. This proposed biomechanical modeling and design approach, integrated with human spinal musculoskeletal characteristics and validated by rigorous simulation and human experiments, provides a generalizable and empirically supported theoretical foundation for the development of rigid-flexible coupled rehabilitation robots across diverse body joints and movement scenarios. The quantitative results fully validate both the device’s performance and the underlying design methodology. Mechanically, the prototype achieved a maximum deformation of 6.40 mm under a 120 N anterior deltoid fascicle force, meeting the design expectations for bionic load buffering; simulation tests yield a maximum lumbar forward flexion angle of 68.6°, lateral flexion angle of 60.4° and rotation angle of 58.5°, all of which closely mirror natural spinal movements of humans; human experiments with ten healthy subjects further confirmed functionality, with average motion angles of 37.6° for forward flexion, 13.56° for backward flexion, 13.62° for lateral flexion and 17.5° for rotation, and an average maximum movement speed of 14.94°/s that nearly aligns with the 14.90°/s design target. All key performance indicators fall close to or within the preset design ranges. Besides validating the effectiveness of the proposed mechanism, the research also establishes a broader methodological contribution: It not only constructs accurate construction of kinematic and static models for rigid-flexible coupled parallel mechanisms but also introduces a standardized analytical framework for flexible component deformation and human-machine motion matching, which can be readily adapted to the design of rehabilitation robots for other joints, such as ankles, knees and upper limbs, thereby filling the theoretical gap in the design of flexible parallel structures for rehabilitation equipment. The findings provide a reliable engineering theoretical basis for the bionic design and performance optimization of such rehabilitation robots. To translate these advances into clinical practice, future work should focus on incorporating adaptive control for patient-specific customization, conducting long-term clinical trials to identify the therapeutic efficacy of the proposed mechanism, and finding lightweight composite materials for improved wearing comfort and suitability for home-based rehabilitation scenarios.

## Data Availability

The original contributions presented in the study are included in the article/supplementary material, further inquiries can be directed to the corresponding author.
